# Supplementary material for: Splice-Junction-Based Mapping of Alternative Isoforms in the Human Proteome
Source: Cell Rep. Author manuscript; Available in PMC 2020 Jan 15. (PMC6961840; doi:10.1016/j.celrep.2019.11.026)
Supplement: 2 [file NIHMS1546469-supplement-2.zip › DF1/PXD006675/SmoothMuscleCells/SmoothMuscleCells_8_PPP1R12A_ISEMEEELKR.pdf]

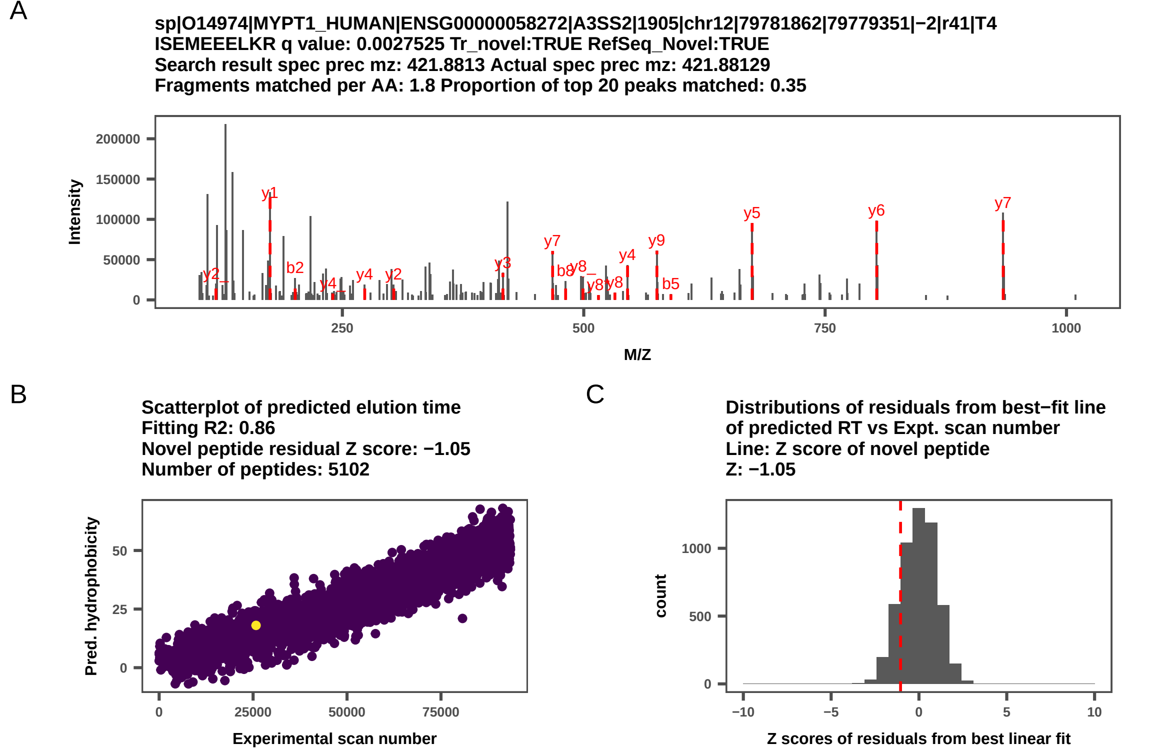
A
sp|O14974|MYPT1_HUMAN|ENSG00000058272|A3SS2|1905|chr12|79781862|79779351|−2|r41|T4
ISEMEEELKR q value: 0.0027525 Tr_novel:TRUE RefSeq_Novel:TRUE
Search result spec prec mz: 421.8813 Actual spec prec mz: 421.88129
Fragments matched per AA: 1.8 Proportion of top 20 peaks matched: 0.35
Intensity
M/Z
B
Scatterplot of predicted elution time
Fitting R2: 0.86
Novel peptide residual Z score: −1.05
Number of peptides: 5102
Pred. hydrophobicity
Experimental scan number
C
Distributions of residuals from best−fit line
of predicted RT vs Expt. scan number
Line: Z score of novel peptide
Z: −1.05
count
Z scores of residuals from best linear fit
